# Antimicrobial Resistance and Molecular Epidemiology of *Corynebacterium striatum* Isolated in a Tertiary Hospital in Turkey

**DOI:** 10.3390/pathogens9020136

**Published:** 2020-02-19

**Authors:** Nergis Asgin, Baris Otlu

**Affiliations:** 1 Department of Medical Microbiology, Faculty of Medicine, Karabuk University, 78100 Karabuk, Turkey; 2 Department of Medical Microbiology, Faculty of Medicine, Inonu University, 44280 Malatya, Turkey; baris.otlu@inonu.edu.tr

**Keywords:** antimicrobial resistance, arbitrarily primed polymerase chain reaction, clone, *Corynebacterium striatum*, molecular epidemiology, nosocomial outbreak

## Abstract

Although *Corynebacterium striatum* is part of the human flora, it has recently drawn attention both for its multidrug resistance and its role as an invasive infection/outbreak agent. This cross-sectional study aimed to determine the antimicrobial resistance and clonal relationships among *C. striatum* strains. In total, 81 *C. striatum* strains were identified using Phoenix-100^TM^ (BD, Sparks, MD, USA). The antimicrobial resistance of the strains was determined using the Kirby–Bauer disk diffusion method. Clonal relatedness among the strains was performed via arbitrarily primed polymerase chain reaction (AP-PCR). All 81 *C. striatum* strains were resistant to penicillin, cefotaxime, ciprofloxacin, and tetracycline, but susceptible to vancomycin and linezolid. The resistance rates to gentamicin, erythromycin, and clindamycin were 34.6%, 79%, and 87.7% respectively. AP-PCR results showed no predominant clone among the *C. striatum* strains. *Corynebacterium striatum* is reportedly the cause of an increasing number of invasive infections/outbreaks. Moreover, treatment options are limited. The study showed that vancomycin, linezolid, and gentamicin can be selected for the empirical treatment of *C. striatum* infections. Although no single-clone outbreak was observed in our hospital, small clonal circulations were observed within some units, indicating cross-contamination. Therefore, a comprehensive infection control program is warranted in future.

## 1. Introduction

The *Corynebacterium* species (spp) are aerobic, non-spore-forming, club-shaped Gram-positive rods. They are ubiquitous in the environment (soil and water) and some species are commensal of normal human skin and mucous membranes. To date, over 100 species have been identified, and 54 of them are associated with human infections [1,2]. Previously, only *Corynebacterium diphtheriae* was considered an infectious agent, whereas other *Corynebacterium* spp. were traditionally dismissed as contaminant bacteria when isolated from clinical specimens. The prevalence of diphtheria has decreased globally after effective vaccination programs. Recently, various non-diphtheriae *Corynebacterium* spp. have been increasingly reported as infectious agents in patients with long-term hospitalization, invasive intervention, and underlying diseases [2,3]. All medically relevant species in the *Corynebacterium* genus are catalase positive and nonmotile. They are divided into lipophilic and nonlipophilic as well as fermentative and nonfermentative. Most of the *Corynebacterium* spp. can be isolated from 5% sheep blood agar. Lipophilic species have enhanced growth in the presence of lipids such as Tween-80. Fermentative species produce acid from various sugars, including glucose, maltose, and sucrose. The most frequently isolated species from clinical samples of humans are *C. jeikeium, C. striatum,* C. *urealyticum,* and *C. amycolatum* [1,4]. *C. jeikeium* and *C. urealyticum* are lipophilic and nonfermentative, whereas *C. striatum* and *C. amycolatum* are nonlipophilic and fermentative [1]. *Corynebacterium jeikeium* is frequently isolated from clinical specimens and has demonstrated nosocomial transmission. They can account for a wide range of human infections, such as respiratory tract infections, septic arthritis, and endocarditis, especially among immunosuppressive patients, including patients with neutropenia, malignancy, and AIDS [2,3]. *Corynebacterium striatum* is the second most commonly isolated *Corynebacterium* species after *C. jeikeium* [5]. Colonies are convex, shiny, moist, and creamy on the medium. The strains produce acid from glucose and reduce nitrate, whereas the Christie-Atkinson-Munch-Peterson (CAMP) test is variable [2].

*Corynebacterium striatum* was first reported in 1980 as the cause of a pleuropulmonary infection in a patient with chronic lymphocytic leukemia [6]. In 1993, the first nosocomial outbreak caused by *C. striatum* was reported, proving that the infection was transmitted from patient to patient via the hands of the hospital staff [7]. Severe invasive infections such as meningitis, blood-stream infections, endocarditis, and osteomyelitis caused by *C. striatum* have been reported in both immunosuppressive and immunocompetent individuals worldwide [8,9,10,11]. Additionally, nosocomial outbreaks due to *C. striatum* have been reported in several countries [12,13]. Moreover, the increase in antimicrobial resistance of *C. striatum* is a great concern. Previously, it was susceptible to many drugs including penicillin, but it has recently demonstrated high-level resistance to antibiotics such as beta-lactams, macrolides, aminoglycosides, and quinolones [14,15,16]. Furthermore, there are some reports on the *C. striatum* strains resistant to daptomycin, which is considered the last-line drug [17,18]. In Turkey, limited data are available on antimicrobial susceptibilities of *C. striatum*, and no study has reported the molecular epidemiology of *C. striatum*. Therefore, we aimed to determine antimicrobial resistance profiles and molecular epidemiology of *C. striatum* isolated from inpatients at a tertiary hospital in Turkey.

## 2. Results

### 2.1. Demographic and Clinical Characteristics of Patients

The demographic and clinical characteristics of the patients from whom strains were obtained from the hospital information system are presented in Table 1.

The mean age of the patients was 71.7 ± 11.7 years (range, 40–95 years) and 76.5% of the patients were aged ≥ 65 years. Of the 81 patients, 42 (51.8%) were male, and 39 (48.2%) were female. The mean age of the male and female was similar and was 69.9 ± 11.4 and 73.6 ± 11.9, respectively *(P* = 0.165). The distributions of patients according to age groups and genders are presented in Table S1. No statistically significant difference was observed between age groups and genders of patients (*P* = 0.446).

Of 81 patients, 68 (84%) patients received treatment in the intensive care unit (ICU), whereas 13 (16%) were treated in the hospital wards. The mean age of patients in the ICU was similar to those in the wards and was 72.7 ± 11.5 and 67.2 ± 11.6, respectively (*P* = 0.134).

All patients had at least one underlying disease. The most common underlying diseases were chronic obstructive pulmonary disease (COPD) (22.2%), heart failure/attack (17.3%), and cerebrovascular disease (14.8%).

Of the 81 *C. striatum* strains, 68 (84%) were isolated from ICUs, and 13 (16%) were isolated from various wards. The strains obtained 35 (43.2%) from blood, 35 (43.2%) from respiratory samples (endotracheal aspirate, sputum, bronchoalveolar lavage fluid), and 11 (13.6%) from wounds.

### 2.2. Antibiotic Resistance Profiles of Isolates

All of the strains (100%) were resistant to penicillin, cefotaxime, ciprofloxacin, and tetracycline, but susceptible to vancomycin and linezolid. The rates of resistance to gentamicin, erythromycin, and clindamycin were 34.6%, 79%, and 87.7%, respectively. The antibiotic resistance rates of strains isolated from ICUs and wards are shown in Table 2. The resistance rates of erythromycin, gentamicin, and clindamycin were similar in the strains isolated from ICUs and wards.

### 2.3. AP-PCR Results

**Figure 1:**
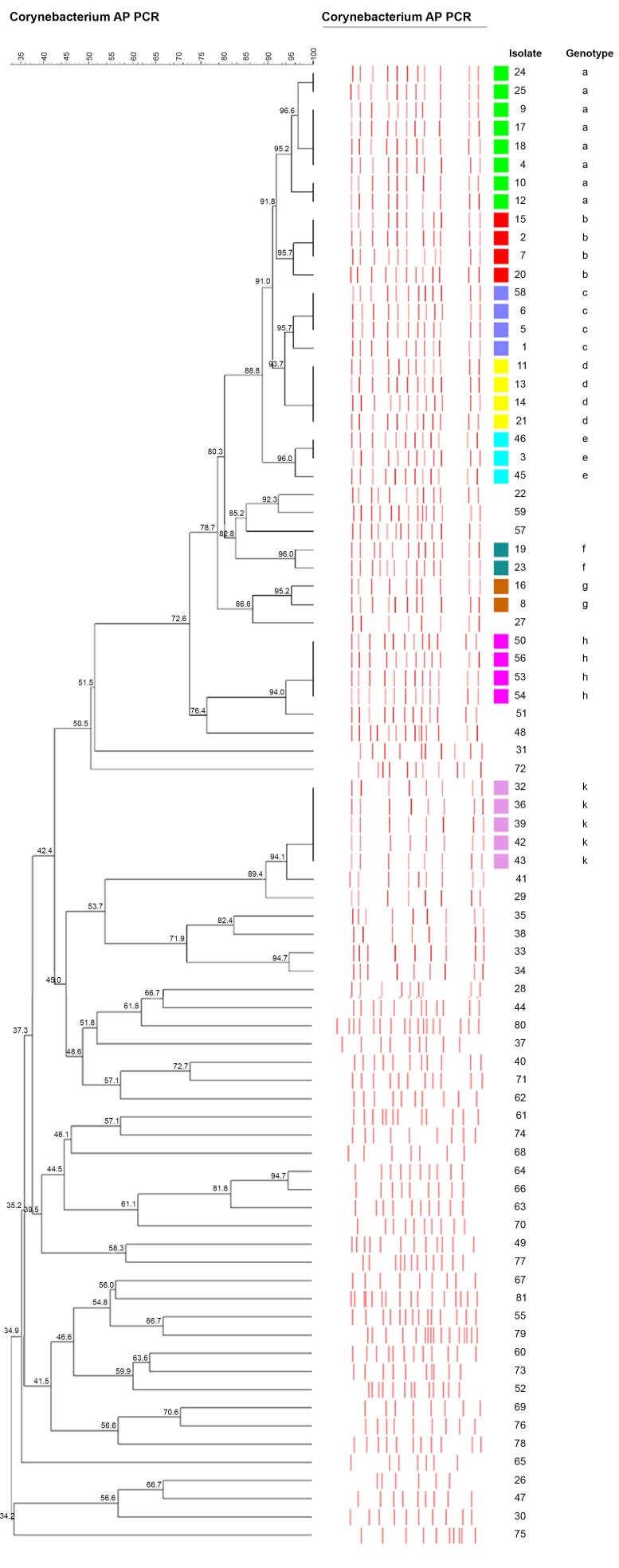
The dendrogram generated according to arbitrarily primed polymerase chain reaction profiles of *Corynebacterium striatum* strains. The clusters were named with lowercase letters: a, b, c, d, e, f, g, h, and k.

The dendrogram generated according to the AP-PCR profiles of the *C. striatum* strains is presented in Figure 1. AP-PCR results showed that there was no predominant clone among the *C. striatum* strains. The 81 *C. striatum* strains showed 54 different profiles, and 36 of the 81 strains were clonally related and formed nine clusters (tolerance: 1.0; cutoff: 95%). Thus, the clustering rate of the strains was 44.4% (36/81). 

The distribution of genotyped and sporadic strains according to the ICU and ward is shown in Table S2. No statistically significant difference was observed between the ICU and ward with respect to genotyped/sporadic strains (*P* = 0.120).

The distribution of genotyped and sporadic strains according to age groups of patients (40–59, 60–79, and >80 years) is shown in Table S3. No statistically significant difference was observed between age groups with respect to genotyped/sporadic strains (*P* = 0.091).

The clusters were named with lower case letters: a, b, c, d, e, f, g, h, and k. The largest cluster was genotype a, which was composed of eight strains and had 10% of the strains. Genotypes k had five strains, and genotypes b, c, d, and h had four strains each. Genotypes e had three strains. The smallest clusters were genotypes f and g, each with two strains.

The characteristics of the 36 *C. striatum* strains genotyped using the AP-PCR method are shown in Table 3.

All 36 genotyped strains were obtained from ICUs and palliative care units. The strains in genotype a (n = 8) were isolated from different ICUs and palliative care units over one year and did not show unique antibiotic resistance profiles. The four strains in genotype b strains were isolated on different dates from various units. They had the same resistance profile and were susceptible to vancomycin, linezolid, and gentamicin. The three strains in genotype e were isolated from respiratory samples obtained from internal medicine ICU over a period of two months. The antibiotic resistance profiles of the strains were the same and were only susceptible to vancomycin and linezolid. The two strains each in genotypes f and g were obtained from the surgical ICU over a period of two months and internal medicine ICU over a period of 10 days, respectively, and were susceptible to vancomycin, linezolid, and gentamicin.

## 3. Discussion

*Corynebacterium striatum* is frequently isolated from patients hospitalized long-term, treated with invasive procedures, and having underlying diseases such as COPD [12,13,19,20]. In this study, the most common underlying disease in patients was COPD, followed by cardiovascular diseases and cerebrovascular diseases. Additionally, the increasing drug resistance in *C. striatum* strains limits treatment options. In earlier reports, *C. striatum* strains were susceptible to many antibiotics [21,22]. However, they have been reported to be multidrug-resistant in recent studies [16,23,24,25]. In the present study, all the *C. striatum* strains were resistant to penicillin, cefotaxime, and ciprofloxacin. In *C. striatum* strains, the *bla* gene that encodes class A beta-lactamase is responsible for penicillin resistance, whereas the *amp C* gene that encodes class C beta-lactamase is responsible for cefotaxime resistance [23]. Quinolone resistance was found to be associated with point mutations in the gyrase subunit A structural gene region [9,26]. In addition, it was reported that antibiotic-resistant mutant strains emerged in bacteria such as *Corynebacterium* spp., which are found in normal flora, following the use of quinolone [23]. A study conducted by Mumcuoglu et al. on 746 *C. striatum* strains isolated in Turkey between 2010 and 2014 demonstrated that the rates of resistance to penicillin and ciprofloxacin were 80% and 83%, respectively [27]. Similarly, Neemuchwala et al. reported high resistance rates (99.9% to penicillin and 95.4% to ciprofloxacin) in 931 *C. striatum* strains isolated in Canada between 2011 and 2016 [28]. Conversely, Alibi et al. [23] from Tunisia reported lower resistance rates (82.5% to penicillin and 36.5% to ciprofloxacin) in 63 *C. striatum* strains isolated between 2011 and 2014. These results may be due to differences in the regional antibiotic resistance profiles of the strains, as well as the different collection dates. Aminoglycoside resistance has been gradually increasing in *C. striatum* strains. The most common mechanism has been reported to be the enzymatic inactivation of the antibiotic molecule [23]. In this study, the resistance to gentamicin was 34.6%, whereas Mumcuoglu et al. [27] reported it to be 17.6%, Neemuchwala et al. [28] as 7.2%, and Alibi et al. [23] as 6.3%. However, Mumcuoglu et al. reported that all 231 (24.8%) strains isolated in 2014 were multidrug-resistant and also resistant to gentamicin [27]. Alibi et al. observed multidrug resistance in 31 (49.2%) of 63 *C. striatum* strains [23]. Wang et al. from China reported that 183 (95.3%) of 192 *C. striatum* strains isolated between 2017 and 2018 were multidrug-resistant and gentamicin resistance rate was 22.9% (44/192) [24]. In the present study, all 81 strains (100%) were resistant to at least three antibiotic groups.

We have found high resistance rates to erythromycin and clindamycin: 79% (64/81) and 87.7% (71/81), respectively. Similarly, Ortiz-Pérez et al. reported resistance rates of 76.4% to erythromycin and 74% to clindamycin in 256 *C. striatum* strains [29]. Resistance to macrolide and lincosamide was reported to be the result of a modification in the region where the antibiotics bind to the methylase enzyme, which is mostly encoded by *erm* (X) genes [26,27,28,29,30]. In this study, all strains (100%) were resistant to tetracycline. Barberis et al. [30] reported tetracycline resistance of 20% (11/55), whereas Wang et al. [24] and Mc Mullen et al [15] reported as 58.3% (112/192) and 62% (128/206) respectively. It has also been reported that the *tetA* and *tetB* genes play a role in the tetracycline resistance of *C. striatum* strains and that the mechanism of resistance involves an active efflux pump [26,31]. Many recent studies have not reported resistance to vancomycin and linezolid [16,23,24,25]. Similarly, in our study, all strains were susceptible to vancomycin and linezolid.

Recently, molecular genotyping methods have been widely used to obtain knowledge about the origin, reservoirs, and circulation patterns of pathogens in case of nosocomial outbreak suspicion. PCR-based molecular typing methods, such as AP-PCR and repetitive element palindromic-PCR (rep-PCR), are faster, easier to use, and easier to interpret than pulsed-field gel electrophoresis (PFGE) which is the gold standard method. Although there is some variability depending on the type of microorganism studied, PCR-based molecular typing methods are reported to have lower discrimination power and reproducibility than PFGE, particularly among closely related strains [32]. In this study, we used the AP-PCR method for molecular epidemiological analysis of the *C. striatum* strains. A total of 36 (44.4%) of 81 strains were clonally related, and nine clusters were formed. As the majority of the clonally related strains were obtained from the surgical and internal medicine ICUs, it was understood that these units were the main sources. The results of AP-PCR revealed no single-clone outbreak at our hospital, but multiclonal spread had taken place. 

Similarly, Mc Mullen et al. [15] reported multiclonal spread among 85 *C. striatum* strains using rep-PCR, isolated between 2013 and 2015 in USA. Although half of the strains were in one group, the remaining strains were distributed into 11 different groups, and the strains in the dominant clone had different antibiotic resistance profiles. Furthermore, they reported that this clonal diversity may be due to the isolation of strains in the non-outbreak period. Alibi et al. [23] detected 22 PFGE profile in 63 *C. striatum* strains from Tunisia and reported high clonal diversity among strains. Wang et al. [19] reported that three epidemic clones were spread by PFGE in 82 *C. striatum* strains isolated from patients’ respiratory samples between 2013 and 2014 in China. Wang et al. [24] applied PFGE to 192 *C. striatum* isolated between 2017 and 2018, and also performed whole genome sequencing in 91 *C. striatum* isolates and reported that there was multiclonal spread within the hospital simultaneously.

In contrary, there are studies reporting single clonal spread in the literature. Baio et al. [33] in 2013 from Brazil and Campanila et al. [31] in 2009 from Italy reported single clonal spread using the PFGE method. Verroken et al. performed both rep-PCR and PFGE methods in 10 outbreak *C. striatum* strains in 2011 in Belgium [13]. Only a single clone was detected by rep-PCR, whereas three distinct types were detected using PFGE. Therefore, they emphasized the high discriminative power of PFGE. 

According to our study results, although there was no single-clone outbreak in our hospital, small clonal circulations were observed within some units. For instance, in small groups, such as genotypes e, f, and g, strains were isolated from the same unit and had the same antibiotic resistance profile and were obtained over a short period. These results indicate cross-contamination. Therefore, we should strictly enforce contact precautions. Hand hygiene is a simple and powerful infection prevention method. Monitoring the use of alcohol-based hand rubs of healthcare workers (HCWs) before and after contact with the patient and encouraging HCWs to remain bare below the elbows are easy and cost-effective measures that can be taken [34,35]. In addition, environmental hygiene should be enhanced. Hospital surfaces and medical equipment should be cleaned with approved disinfectants at appropriate concentration and times.

To the best of our knowledge, this is the first report on the molecular epidemiological analysis of *C. striatum* clinical isolates in Turkey. Of course, there are some limitations. First, this was a retrospective and monocenter study. Second, the *C. striatum* strains were obtained during routine laboratory work and were not accompanied by clinical data, antibiotic treatment regimens, or whether the strains were colonizers or causes of infection. Third, the AP-PCR method was used for molecular epidemiological analysis of the isolates instead of the PFGE. PFGE is a labor-intensive method that requires experienced staff and special equipment. AP-PCR is a more practical method because it is easy and cheap to perform and obtained the results within a short time. Therefore, AP-PCR can be performed during an outbreak, allowing the source of the outbreak to be rapidly detected.

## 4. Materials and Methods

### 4.1. Study Design

This cross-sectional study was conducted between November 2018-February 2019 at the Karabuk University Training and Research Hospital, a 440-bed tertiary hospital in the city of Karabuk, western Black Sea region of Turkey.

### 4.2. Isolation and Identification of C. striatum Strains

The study included 81 *C. striatum* strains isolated from routine clinical samples of inpatients at Karabuk University Training and Research Hospital between January 2015 and February 2016.

The strains were stored in brain-hearth infusion broth with 20% glycerol at −80 °C until use. Only one strain from each patient was included. Repetitive, other *Corynebacterium* species, contaminated, or unanimated strains were excluded.

Clinical samples (blood, wound, endotracheal aspirate, bronchoalveolar lavage fluid, sputum) collected from inpatients were inoculated on 5% sheep blood Columbia agar [Becton Dickinson and Company (BD), Sparks, MD, USA], eosin methylene blue (EMB) agar (BD), and chocolate agar (BD). For blood culture, 8–10 mL of each patient’s blood was inoculated into BD BACTEC Plus vials and incubated in the Bactec FX 40 (BD, MD, USA) automated blood culture system for seven days. Samples with positive signals were inoculated on 5% sheep blood Columbia agar, EMB agar, and chocolate agar plates. After an incubation period of 24–48 h, 35°C in 5% CO_2_ atmosphere conditions, Gram staining was performed on the catalase-positive colonies and microscopically examined. When Gram-positive pleomorphic bacilli were seen, the colonies were identified using the BD Phoenix^TM^ automated system. Identification of isolates was confirmed using the matrix-assisted laser desorption/ionization time-of-flight (MALDI-TOF) method with the VITEK®-MS device (BioMérieux, Marcy-l’Étoile, France) at the Inonu University School of Medicine, Molecular Microbiology Laboratory in Malatya.

### 4.3. Antibiotic Susceptibility Testing

The antibiotic susceptibility of the strains was determined using the Kirby–Bauer disk diffusion method. The bacterial suspension adjusted 0.5 Mc Farland turbidity was inoculated by sterile cotton swap to the surface of Mueller–Hinton fastidious agar (MH-F, BD, MD, USA) plate containing 5% defibrinated horse blood and 20 mg/L β-NAD. Nine antibiotic discs were used: Penicillin (1 unit), ciprofloxacin (5 µg), gentamicin (10 µg), vancomycin (5 µg), linezolid (10 µg), erythromycin (15 µg), clindamycin (2 µg), cefotaxime (5 µg), and tetracycline (30 µg). Antibiotic discs were provided by BBL (BD, Sparks MD, USA) and MAST (MAST Group, Liverpool, UK). The antibiotic discs were placed on the MH-F agar plates within 15 min of inoculation on the plates. The plates were incubated for 16–24 h at 35 °C in 5% CO_2_ atmosphere conditions. The inhibition zone diameters formed around the antibiotic discs were measured and interpreted using the European Committee on Antimicrobial Susceptibility Testing (EUCAST) guidelines [36]. However, because the erythromycin zone diameter breakpoint is not available for *Corynebacterium* spp. in the EUCAST guidelines, the EUCAST interpretive criteria for *Streptococcus pneumoniae* was used. Accordingly, *C. striatum* strains showing an inhibition zone diameter of ≥22 mm were considered sensitive and <19 mm were considered resistant. Similarly, the zone diameter breakpoint recommended by EUCAST for Viridans streptococci was used for cefotaxime. As such, *C. striatum* strains showing an inhibition zone diameter of ≥23 mm were considered susceptible and <23 mm were considered resistant. *Streptococcus pneumoniae* ATCC 49619 was used for quality control [36]. The antibiotic susceptibility results of strains were recorded.

### 4.4. Determination of the Clonal Relationship among C. striatum Strains

The clonal relationship among *C. striatum* strains was determined using the arbitrarily primed polymerase chain reaction (AP-PCR) method. AP-PCR was carried out at the Inonu University Molecular Microbiology Laboratory as described previously [37]. The stock cultures of *C. striatum* strains were inoculated on 5% sheep blood agar. After 24–48 h of incubation, pure bacterial cultures were obtained. Genomic DNA was isolated using the QIAsymphony total nucleic acid isolation kit. M13 primers (5′-GAGGGTGGCGGTTCT-3′) were used for amplification under the following conditions: Initial denaturation for 5 min at 94 °C, followed by 40 cycles consisting of denaturation at 94 °C for 60 s, annealing at 40 °C for 60 s, and extension at 72 °C for 2 min. The amplification products were separated by electrophoresis in a 1.5% agarose gel with ethidium bromide for 1.5 h at 100 V, and the band profiles were visualized using the Gel Logic 2200 imaging system (Kodak Co., Rochester, NY, USA) under ultraviolet light. The band profiles were analyzed using the Gel Compar version 6.6 software program (Applied Maths, Kortrijk, Belgium). The Dice Similarity Coefficient (DSC) was used in similarity calculations for band analysis, whereas the Unweighted Pairwise Grouping Mathematical Averaging (UPGMA) method was used for clustering analysis. The strains were identified as different genotypes if the DSC was <95% and as the same genotype if it was ≥95%. A dendrogram was created using the UPGMA clustering algorithm.

### 4.5. Statistical Analysis

The data were analyzed using the Minitab 17 (Minitab, Inc., PA, USA) statistical software program. Descriptive statistics were expressed as number (n), percentage (%), and mean and standard deviation. The Kolmogorov–Smirnov test was performed to determine whether the variables were normally distributed. For the comparison of continuous variables, two-sample t-test was used. The Pearson’s Chi-squared test or Fisher’s Exact test was used for comparison of categorical variables if applicable. A probability *(P)* value of ≤0.05 was considered statistically significant in the 95% confidence interval.

### 4.6. Ethical Approval

This study was reviewed and approved by the Noninterventional Clinical Research Ethics Board of Karabuk University (Date: October 31, 2018; decision no: 10/11).

## 5. Conclusions

This study highlighted the increasingly frequent occurrence of *C. striatum* as an invasive infection/outbreak agent and the rising antibiotic resistance of strains. Although *C. striatum* is part of the normal human flora, it should not be overlooked when isolated from clinical specimens, as it may actually be a cause of infection. Therefore, the microbiologist and clinician should cooperate to reach a final decision. In the present study, *C. striatum* strains showed high-level resistance to most commonly used antibiotics. Therefore, based on our *in-vitro* antibiotic susceptibilities results, vancomycin, linezolid, and gentamicin can be selected for the empirical treatment of *C. striatum* infections. However, the antibiotic resistance profile of *C. striatum* should be monitored regularly, and empirical antibiotic treatment regimens should be revised. Although no single-clone *C. striatum* outbreak was detected in our hospital, polyclonal dissemination was detected and small clonal spreads were observed, indicating cross-contamination. Therefore, we should perform the effective infection control measures. In addition, compliance of HCWs to hand hygiene and cleaning of medical equipment and hospital surfaces with appropriate disinfectant should be monitored.

## Figures and Tables

**Table 1:** Demographic and clinical features of patients (n = 81)

Data	Number (%)
**Age (years)**	
40–59	12 (14.8)
60–79	42 (51.9)
≥80	27 (33.3)
**Gender**	
Female	39 (48.1)
Male	42 (51.9)
**Hospital Unit**	
**Intensive Care Unit (ICU)**	68 (84.0)
Surgical ICU	23 (28.4)
İnternal medicine ICU	22 (27.2)
Reanimation ICU	16 (19.8)
Coronary ICU	7 (8.6)
**Wards**	13 (16.0)
General surgery	5 (6.2)
Palliative care	4 (4.9)
Respiratory	4 (4.9)
**Specimens**	
Blood	35 (43.2)
Endotracheal aspirate	24 (29.6)
Wound	11 (13.6)
Sputum	7 (8.7)
Bronchoalveolar lavage fluid	4 (4.9)
**Underlying Diseases**	
*COPD	18 (22.2)
Heart failure/ attack	14 (17.3)
Cerebrovascular disease	12 (14.8)
Cardiac arrest	9 (11.1)
Renal failure	9 (11.1)
Acute abdomen with ileus	8 (9.9)
Surgical site infection	7 (8.6)
Sepsis	5 (6.2)
Gastrointestinal hemorrhage	4 (4.9)
Traumatic pneumothorax	4 (4.9)
Femur fracture	3 (3.7)
Hepatobiliary diseases	3 (3.7)

* Chronic obstructive pulmonary disease.

**Table 2:** Antibiotic resistance rates of *Corynebacterium striatum* strains isolated from the ICUs and wards.

	Total (n = 81)	ICUs (n = 68)	Wards (n = 13)	*P*-value
**Antibiotic**	n (%)	n (%)	n (%)	
Erythromycin	64 (79)	55 (80.8)	9 (69.2)	0.363
Clindamycin	71 (87.7)	59 (86.6)	12 (92.3)	0.578
Gentamicin	28 (34.6)	23 (33.8)	5 (38.5)	0.747
Ciprofloxacin	81 (100)	68 (100)	13 (100)	Na
Penicillin	81 (100)	68 (100)	13 (100)	Na
Cefotaxime	81 (100)	68 (100)	13 (100)	Na
Ciprofloxacin	81 (100)	68 (100)	13 (100)	Na
Tetracycline	81 (100)	68 (100)	13 (100)	Na
Vancomycin	0 (0)	0 (0)	0 (0)	Na
Linezolid	0 (0)	0 (0)	0 (0)	Na

**Abbreviations**: ICU: Intensive care unit; Na: Not applicable.

**Table 3:** Characteristics of *Corynebacterium striatum* strains genotyped by arbitrarily primed polymerase chain reaction (n = 36).

Strain no	Department	Source	Isolation Date	Antibiotic Susceptibility	Genotype
4	surgical ICU	EA	13.4.2015	Va, Lzd	a
9	internal medicine ICU	EA	18.4.2015	Va, Lzd, Gn, E	a
10	reanimation ICU	EA	8.2.2016	Va, Lzd	a
12	surgical ICU	Blood	2.10.2015	Va, Lzd	a
17	internal medicine ICU	EA	4.10.2015	Va, Lzd, Gn, E, Da	a
18	palliative care unit	wound	27.12.2015	Va, Lzd, E	a
24	coronary ICU	sputum	21.11.2015	Va, Lzd	a
25	surgical ICU	wound	7.2.2015	Va, Lzd	a
2	surgical ICU	Blood	8.4.2015	Va, Lzd, Gn	b
7	internal medicine ICU	EA	19.10.2015	Va, Lzd, Gn	b
15	reanimation ICU	EA	10.1.2015	Va, Lzd, Gn	b
20	palliative care unit	sputum	13.12.2015	Va, Lzd, Gn	b
58	internal medicine ICU	BLF	11.5.2015	Va, Lzd, Gn	c
1	surgical ICU	Blood	30.1.2015	Va, Lzd, Gn	c
5	reanimation ICU	EA	7.2.2015	Va, Lzd, Gn	c
6	internal medicine ICU	Blood	29.5.2015	Va, Lzd, E	c
11	internal medicine ICU	Blood	5.5.2015	Va, Lzd, Gn	d
13	surgical ICU	EA	23.10.2015	Va, Lzd, Gn	d
14	internal medicine ICU	BLF	26.8.2015	Va, Lzd	d
21	surgical ICU	EA	14.1.2016	Va, Lzd	d
3	internal medicine ICU	EA	4.1.2016	Va, Lzd	e
45	internal medicine ICU	sputum	6.11.2015	Va, Lzd	e
46	internal medicine ICU	EA	4.12.2015	Va, Lzd	e
19	surgical ICU	wound	17.4.2015	Va, Lzd, Gn	f
23	surgical ICU	sputum	4.2.2015	Va, Lzd, Gn	f
8	internal medicine ICU	BLF	3.4.2015	Va, Lzd, Gn	g
16	internal medicine ICU	Blood	24.3.2015	Va, Lzd, Gn	g
50	surgical ICU	Blood	24.7.2015	Va, Lzd, Gn	h
53	surgical ICU	Blood	1.4.2015	Va, Lzd, Gn	h
54	coronary ICU	EA	8.1.2015	Va, Lzd, Gn, E, Da	h
56	coronary ICU	sputum	24.2.2015	Va, Lzd, Gn	h
32	surgical ICU	EA	21.12.2015	Va, Lzd	k
36	reanimation ICU	Blood	23.5.2015	Va, Lzd, Gn, E, Da	k
39	reanimation ICU	EA	4.7.2015	Va, Lzd	k
42	surgical ICU	EA	20.5.2015	Va, Lzd, Gn	k
43	reanimation ICU	BLF	23.11.2015	Va, Lzd, Gn, E	k

**Abbreviations:** ICU: Intensive care unit, EA: Endotracheal aspirate, BLF: Bronchoalveolar lavage fluid. Va: Vancomycin, Lzd: Linezolid, Gn: Gentamicin, E: Erythromycin, Da: Clindamycin.

## Supplementary Materials

The following are available online at https://www.mdpi.com/2076-0817/9/2/136/s1, Table S1: Distributions of *C. striatum* strains isolated from patients according to the age groups and gender; Table S2: Distributions of genotyped and sporadic *C. striatum* strains according to intensive care units and wards. Table S3: Distribution of genotyped and sporadic *C. striatum* strains according to the age groups.
