# Supplementary material for: Antimicrobial Resistance and Molecular Epidemiology of Corynebacterium striatum Isolated in a Tertiary Hospital in Turkey
Source: Pathogens. 2020 Feb 19;9(2):136. doi: 10.3390/pathogens9020136 (PMC7168331; doi:10.3390/pathogens9020136)
Supplement: Supplementary file 1 [file pathogens-09-00136-s001.pdf]

Table S1. Distributions of C. *striatum* strains isolated from patients according to the age groups and genders n (%)

Age groups (years)	Female (n = 39)	Male (n = 42)	Total
40–59	5 (12.8)	7 (16.7)	12(14.8)
60–79	18 (46.2)	24 (57.1)	42 (51.9)
≥80	16 (41)	11 (26.2)	27(33.3)
Total	39(100)	42 (100)	81(100)

P = 0.366 (Pearson's chi-squared test)

Table S2. Distributions of genotyped and sporadic *C. striatum* strains according to intensive care units and wards n (%)

Strains	Intensive care units (n = 68)	Wards (n = 13)	Total
Genotyped	33 (48.5)	3 (23.1)	36 (44.4)
Sporadic	35 (51.5)	10(76.9)	45 (55.6)
Total	68 (100)	13 (100)	81 (100)

P = 0.091 (Fisher's exact test)

Table S3: Distribution of genotyped and sporadic *C. striatum* strains according to the age groups n (%)

Age groups (years)	Genotyped (n = 36)	Sporadic (n = 45)	Total
40–59	3 (8.3)	9 (20)	12(14.8)
60–79	23 (63.9)	19 (42.2)	42 (51.9)
≥80	10 (27.8)	17 (37.8)	27(33.3)
Total	36 (100)	45 (100)	81(100)

P = 0.120 (Fisher's exact test)
